# Using community health workers to refer pregnant women and young children to health care facilities in rural West Bengal, India: A prospective cohort study

**DOI:** 10.1371/journal.pone.0199607

**Published:** 2018-06-21

**Authors:** Abram L. Wagner, Lu Xia, Aparna Ghosh, Sandip Datta, Priyamvada Pandey, Sujay Santra, Sharmila Chattopadhyay, Uddip Nandi, Tanusree Mazumder, Sucheta Joshi, Joyojeet Pal, Bhramar Mukherjee

**Affiliations:** 1 Department of Epidemiology, School of Public Health, University of Michigan, Ann Arbor, Michigan, United States of America; 2 Department of Biostatistics, School of Public Health, University of Michigan, Ann Arbor, Michigan, United States of America; 3 iKure Techsoft, Kolkata, West Bengal, India; 4 Center for Global Health and Development, University of Nebraska Medical Center, College of Public Health, Omaha, Nebraska, United States of America; 5 Department of Pediatrics and Communicable Diseases, Pediatric Neurology, University of Michigan Medical School, Ann Arbor, Michigan, United States of America; 6 School of Information, University of Michigan, Ann Arbor, Michigan, United States of America; Nathan S Kline Institute, UNITED STATES

## Abstract

**Background:**

Community health workers (CHWs) have been placed in many rural areas in India to increase villagers’ connections to basic preventive health care. In this study, we describe how pregnant women and mothers of young children react when CHWs inform them that they, or their child, are at high risk of pregnancy-related complications or early childhood developmental delays, and further screening and health care from a physician is recommended.

**Methods:**

In this longitudinal study in rural villages in West Bengal, India, pregnant mothers, as well as mothers of children aged 12–24 months, were screened for high risk complications. They were re-contacted and asked questions regarding how and to what extent did visits by the CHWs improve their household’s overall health behavior, along with details about what additional care, if any, they sought. These responses are presented by different demographic and medical characteristics.

**Results:**

Of the 231 pregnant women, all said they had sought additional care in response to the CHW visit, and all stated that feedback from the CHW resulted in improvement to their health behaviors. Most (90%) pregnant women gave birth at an institution. Among the 213 mothers of young children who were followed up, all sought additional care in response to the CHW’s visit. Most (67%) mentioned that they had a significant improvement in their health behaviors following feedback from the CHW, and the rest stated that they had some improvement.

**Conclusions:**

With the proper training, CHWs can be partners in health care to improve the health of vulnerable populations, not only in rural areas of India, but also in other developing countries. CHWs can promote positive health outcomes in their villages of residence.

## Introduction

Community health workers (CHWs) have been an integral part of the health care system throughout the world since the 1970s, especially in low- and middle-income countries [1]. They often work with marginalized populations in medically underserved areas that lack consistent access to health care services. Their role is to increase awareness and usage of preventive care (e.g., management of chronic diseases and access to antenatal services), to enhance provider-patient communication, to monitor health status or adherence to treatment, and to provide a link between patients and health or other social services [2]. CHWs could play an important role in countering maternal and child morbidity. Currently, up to 50% of women may experience pregnancy-related complications [3], and around 10% of children globally may experience developmental disabilities [4]. These numbers appear to be increasing [5], and the World Health Organization (WHO) has established guidelines to help curb this phenomenon [6].

Improved access to preventive care in India, particularly in rural areas, could be beneficial to countering high rates of maternal and child morbidity; India accounts for 15% of maternal deaths globally (45,000, or 174 deaths per 100,000 live births in 2015) [7]. As of 2010, the infant mortality rate was 47 deaths per 1,000 live births (51 per 1,000 in rural areas vs 31 per 1,000 in urban areas) [8]. Many of the tools currently available for screening have been developed in Western countries, but have not been studied in depth in other countries [9]. Low and middle- income counties often have trouble implementing preventive measures, especially in rural areas and areas where socioeconomically disadvantaged people live. In India, these areas tend to be more remote, medically underserved, and consistently understaffed [10].

The Indian government has worked to fix this urban-rural gap by starting various initiatives to improve health outcomes in rural and remote areas, particularly through promoting the use of CHWs. Currently almost half of CHWs in the world reside in India [1]. While previous studies have shown that utilizing CHWs in their villages provides them leadership opportunities and positively impacts the health of the community, there is little research in the public health community on the feasibility of utilizing CHWs to conduct research studies, especially in conjunction with smart devices, used both to collect the data securely into the cloud, and to screen individuals using automated algorithms.

Previously, we examined the demographic and health profile of mothers who were at high risk for complications during pregnancy (low body weight, low hemoglobin (<11 g/dl), or low or high blood pressure) and young children who were at risk for developmental delays (abnormal head circumference, upper arm circumference, or weight percentiles; or low score from the Ages and Stages Questionnaire (ASQ)) [11]. We enrolled individuals from rural West Bengal state, in West Midnapore district, which is part of India’s Backwards Region Grant Programme, because of its underperformance on medical and socioeconomic measures [12]. In the district’s rural regions, 21.9% of births occur at home, and only 41.1% of pregnant women receive the full complement of antenatal care [13]. In this study, we describe how pregnant women and mothers of young children react when presented with information that they, or their child, is at high risk and is indicated for further screening and health care from a physician. This push for a visit with a trained medical provider could be one mechanism to increase the proportion of individuals who have access to health care services, and to decrease morbidity from preventable causes among women and infants. We present findings from this study as a guide for future studies, which may formally compare the effectiveness of screening programs with or without CHWs or with or without smart devices.

## Materials and methods

### Study population

This longitudinal study was conducted from July 2015 to January 2016 in Debra block, which is a rural area in West Midnapore district, West Bengal state [14]. There are 476 villages in Debra; 60 of them are currently sites of health promotion activities from the social enterprise iKure, and 19 of these were randomly chosen as the sampling frame for this study. Ten CHWs were trained by one medical doctor and two research personnel to conduct the study. Within a six-hour training session, the CHWs were shown how to take blood pressure, arm and head circumferences, and hemoglobin. CHWs were also trained on how to use a mobile device and on informed consent.

Our sample size is based off the aims of a previous study [11]: calculating a 5% margin of error around the prevalence of high risk conditions. Based off previous studies in India, we estimated that 21% of women would have a pregnancy-related complication [15] (necessitating a sample size of 255) and 51% of children would have low scores on at least one ASQ domain [16](necessitating a sample size of 383). Convenience samples of pregnant mothers (range: 4–32 per village) and mothers of young children (range: 6–52 per village) who attended these health promotion events were enrolled in the initial study. As reported in a previous study [11], these participants were screened for high risk conditions, and women with a high risk pregnancy and mothers of children who had indications of a developmental delay were referred to health care facilities.

The CHW interviewers used smart devices—either a tablet or smart phone—to collect data and were equipped with a blood pressure machine, a weighing machine, a measuring tape, and a blood testing kit (Hemocheck, Allied Healthsciences, New Delhi, India). Pregnant women were considered at risk if they were anemic (hemoglobin concentration <11 g/dL), underweight (<40 kg), or had low (<110/70 mmHg) or high (>140/90 mmHg) blood pressure. For young children, indications of potential developmental delays included abnormal head circumference (<22^nd^ or >95^th^ percentile), upper arm circumference, and weight (outside the 3^rd^ and 97^th^ percentiles according to WHO child growth standards [17]). Young children were also referred to a doctor if they had low scores (i.e., 2 standard deviations below US standards) on a domain of the ASQ, which had been translated from English to Bengali for the purposes of this study. The CHWs screened participants for high risk conditions in their homes using an Open Data Kit (ODK) Collect Application on a smart device [18], which digitized the forms for maternal and child health evaluation. The participants considered high risk were referred to health care services and were later re-contacted (the messaging used is available in S1 Appendix): pregnant women within 60 days of their due date, and children 3 to 6 months after the previous visit.

The outcome of this study was participants’ behavior after being contacted about a potential health issue. The primary outcome was self-rated improvement in health behavior. All participants were asked to what extent did visits by the community health worker improve their household’s overall health behavior (none, some, or significant improvement). As a secondary outcome, participants were asked about what steps they took based on the CHWs’ information. Pregnant women were asked where they gave birth (home, government hospital, or private hospital) and how feedback provided by the community health worker changed their health behavior (sought additional care, changed diet and physical activity, modified other health behaviors, or no appreciable change). Mothers of young children were asked if they sought pediatric care for their child’s developmental issues based on the community health worker feedback (yes, not yet, or do not intend to).

The study presents a description of outcomes by different demographic and medical characteristics. The main explanatory variables were age of mother, number of children in household, education, and income. Data were analyzed in SAS version 9.4 (SAS Institute, Cary, NC, USA).

### Ethical approval

This study was approved by the University of Michigan Health Sciences and Behavioral Sciences Institutional Review Board (study number HUM00099965) and an institutional ethical committee at iKure. The pregnant mothers and mothers of young children gave written informed consent before any data were collected. As an incentive for their participation, pregnant mothers were given nutrient supplements and the young children were given toys. All participants also received their screening results.

## Results

**Fig 1:**
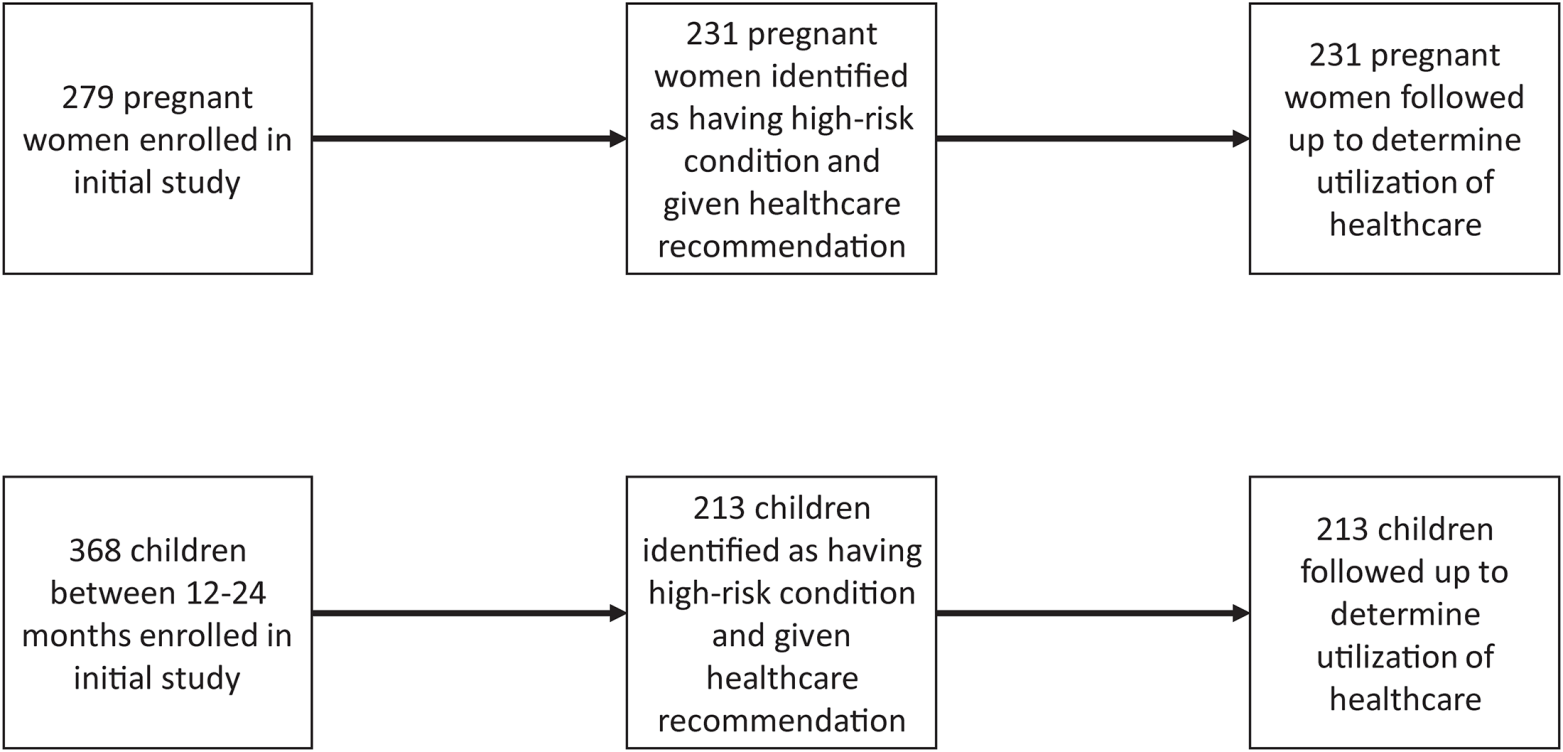
Selection of mothers and children into the study.

Initially, 279 pregnant women and 368 children were enrolled in the study. Of these, 231 (83%) of the pregnant women and 213 of the children (58%) were referred to a health care facility because of a high-risk condition. The most common pregnancy high-risk conditions were anemia—in 49% of the women, low blood pressure—in 35% of women, and low body weight—in 8% of the women; among children, 50% had an abnormal result from at least one of the anthropometric tests, and 24% had low scores on at least one domain of the ASQ. Demographic characteristics of the participants have been presented in a previous paper [11]. The study design is depicted in Fig 1.

Of the 231 pregnant women who were followed up, 6 (3%) experienced miscarriages or stillbirths. All pregnant women said they had sought additional care in response to the CHW visit, and all stated that feedback from the CHW resulted in significant improvement to their health behaviors. Most pregnant women either gave birth at a government institution (46%) or a private institution (45%), with only 10% delivering at home (Table 1). There were not large differences in place of delivery across different demographic or medical groups, but women with more children were more likely to deliver at home than women with fewer children (for example, 17% of women with 2–4 children delivered at home, compared to 6% with no other children). When the head of household was illiterate, the pregnant woman was more likely to deliver at home (17%) than if the head of household had even a primary school education (6%).

**Table 1:** The distribution of demographic and medical characteristics, and responses to a question about where the pregnant women gave birth, among 231 women with at-risk conditions during pregnancy in rural West Bengal, India, 2015–16.

		Home delivery	Delivery at a government institution	Delivery at a private institution
**Overall**		22 (10%)	106 (46%)	103 (45%)
**Age of mother**	16–19 years	4 (6%)	31 (45%)	34 (49%)
	20–24 years	11 (10%)	52 (45%)	52 (45%)
	25–37 years	7 (15%)	23 (49%)	17 (36%)
**Number of children**	0	8 (6%)	54 (43%)	65 (51%)
	1	10 (12%)	37 (46%)	34 (42%)
	2–4	4 (17%)	15 (65%)	4 (17%)
**Number of antenatal visits**	0–1	2 (7%)	13 (46%)	13 (46%)
	2	6 (11%)	24 (45%)	23 (43%)
	3	5 (7%)	33 (45%)	35 (48%)
	4	5 (9%)	23 (43%)	26 (48%)
	5–8	4 (17%)	13 (57%)	6 (26%)
**Head of household’s education**	Illiterate	4 (17%)	12 (50%)	8 (33%)
	Primary school	7 (6%)	61 (54%)	45 (40%)
	High school and above	11 (12%)	33 (35%)	50 (53%)
**Source of household income**	Agriculture	5 (9%)	19 (36%)	29 (55%)
	Employment or small business	1 (5%)	7 (32%)	14 (64%)
	Daily wage	16 (10%)	80 (51%)	60 (38%)
**Monthly income**	<5,000 Rupees	16 (10%)	80 (50%)	64 (40%)
	≥5,001 Rupees	6 (8%)	26 (37%)	39 (55%)
**Family type**	Nuclear	8 (9%)	51 (57%)	31 (34%)
	Joint	14 (10%)	55 (39%)	72 (51%)
**Abnormal weight in mother**	No	19 (9%)	95 (45%)	99 (46%)
	Yes	3 (17%)	11 (61%)	4 (22%)
**Abnormal blood pressure in mother**	No	15 (10%)	65 (44%)	68 (46%)
	Yes	7 (9%)	41 (50%)	34 (41%)
**Anemia in mother**	No	15 (12%)	52 (40%)	62 (48%)
	Yes	7 (7%)	54 (53%)	41 (40%)

Among the 213 mothers of young children who were followed up, all sought additional care in response to the CHW’s visit. Two-thirds mentioned that they had a significant improvement in their health behaviors following feedback from the CHW, and the rest stated that they had some improvement (Table 2). The mothers who delivered at home, or had not visited child specialists before, reported higher significant improvement rate than average (76% and 77%, respectively).

**Table 2:** Stated perceptions of improvement in overall health behavior (no, some, or significant improvement) after receiving visits from community health workers among 213 mothers of children with indications for health care need in rural West Bengal, 2015–16.

		Some improvement	Significant improvement
**Overall**		71 (33%)	142 (67%)
**Birth weight of child**	Normal	58 (32%)	123 (68%)
	Low	13 (41%)	19 (59%)
**Gestational age at child’s birth**	Full term	55 (34%)	106 (66%)
	Premature	16 (31%)	36 (69%)
**Sex of child**	Male	28 (27%)	77 (73%)
	Female	43 (40%)	65 (60%)
**Child’s place of birth**	Home	7 (24%)	22 (76%)
	Institution	64 (35%)	120 (65%)
**Visits a child specialist**	No	12 (23%)	41 (77%)
	Yes	59 (37%)	101 (63%)
**Low Ages and Stages Questionnaire score**	No	41 (32%)	87 (68%)
	Yes	30 (35%)	55 (65%)
**Abnormal anthropometric measure**	No	17 (45%)	21 (55%)
	Yes	54 (31%)	121 (69%)

## Discussion

Health screening is not a current health care priority in India and only in the last two decades has there been a gradual increase in the focus on early screening, i.e. in the first trimester of pregnancy [19]. Given myriad health issues facing those specifically living in rural areas, elevating the importance of screening is imperative to improving maternal and infant mortality outcomes in India. This study re-contacted pregnant women and young children who had been notified that they had a high-risk condition, and found that most stated that feedback from the CHW resulted in improvement to their health behaviors, and most made changes or sought additional care.

In this feasibility study, we showed specifically how CHWs can implement screening programs and directly improve health outcomes of women during pregnancy and children early in their development. Even though they had limited prior exposure to this technology, the CHWs used smart devices to screen women for complications during pregnancy and young children for developmental delays. The participants in the study were comfortable with the process of being screened by the CHWs, and actively worked to change health behaviors as a direct result of feedback from the CHWs. For example, although we did not have a formal “control” group, we saw a high prevalence of institutional delivery in this sample as opposed to high prevalence of home delivery in the surrounding district at-large [13].

### CHWs’ use of smart devices

The use of smart devices is a promising tool for future programs to increase the use of health services among pregnant women and among young children; for the latter group, this is particularly important after their second year, when they are still rapidly developing but when they may not be consistently receiving well-child visits alongside regular vaccination visits. Sending CHWs into the community to conduct simple screenings may be an efficient and cost-effective way of promoting health within these communities [20]. There are other common applications of mHealth, including educating clients and encouraging behavior change and improving efficiency of the work of health care providers—for instance through provider-to-provider communication, planning and scheduling of provider work, and training programs [21].

Encouraging CHWs to utilize smart devices has been a strategy employed in other low- and middle-income countries [22], and we suspect that smart devices can catalyze the effectiveness of future programs that use CHWs through several mechanisms: (1) data collected on smart devices can be automatically synched to the cloud and can mitigate errors in data collection, (2) data collection applications can support translation into multiple languages, which can be helpful for CHWs facing diverse and multilingual villages, (3) an algorithm on the device can be automatically employed to screen for high-risk health conditions, and the CHW can provide direct feedback to the family after the interview. Additionally, findings from the CHW visit could be automatically forwarded to a health center, especially if the family has a prior relation with a health center. However, there are some potential limitations to the use of smart devices. The effectiveness of CHWs using smart devices can be limited by several factors, including the agency or self-efficacy of CHWs within an organization, the amount of training that the CHW has received, and the comfort that the data collectors have with the project itself [23].

Smart devices, and applications like Dimagi’s CommCare, have emerged as a potential solution to insufficient numbers of skilled health care workers in remote areas of India and other low- and middle- and low-income countries [24,25]. Kallander et al. summarize findings from research in the mobile health sphere as an effective way to provide health care services to all communities [22]. Most previous studies have focused on families having smart devices and receiving a one-way communication from the doctor, for example about upcoming appointments [22]. However, this use of mobile phones is limited in many areas in India; although the proportion of rural individuals in India who have mobile phones has increased from 22% to 38% between 2010 and 2014 [26], a majority of the rural population does not have access to this technology. Using CHWs with smart devices as a messenger of health information, instead of the smart devices themselves, removes problems related to access to mobile phones in rural India.

A future study should therefore be designed to formally test the effectiveness of CHWs using smart devices on the health of a community. For example, within a village, families could be randomized into two groups: one group having a CHW who uses a smart device, the other group having a CHW without a smart device. That study could also tease out any difficulties that CHWs have in using the smart device, and could improve our understanding of how CHWs are able to use technology to navigate complex linguistic and cultural issues in their communities.

### Strengths and limitations

As this is a feasibility study with a limited number of participants, there were a few limitations that could incur bias. Because the women had previously dealt with the same CHWs in a previous wave of data collection, they may have over-reported desirable behaviors (i.e., being satisfied with the CHWs or stating that they received health care services). All of the work conducted in this study is self-reported, with no verification of hospital records. The study is also not overly generalizable, as the rural areas around Kolkata alone differ based on geography, language, religion, and other cultural factors. Additionally, by selecting a convenience sample we may have a sample biased towards having relatively health behaviors or being more willing to participate in studies. We did not have a large enough sample size to test for confounding, but future studies could look at the role of socioeconomic status and other potential confounders on the relationship between messaging from CHWs and changes in health care behaviors.

However, we were able to conduct the study using a cohort design, and we used a validated tool—the ASQ—which has been previously used (in translation) in India [16]. We also had a set group of CHWs and a set group of women who were enrolled in the study, so the participants and the CHWs were able to build a level of trust and rapport with one another.

### Conclusions

Based on our work in the rural areas of Kolkata, India, health care provision and outcomes are poor and interventions are needed to better help rural residents. By training CHWs to use smart devices to screen for pregnancy complications and developmental delays in young children, the CHWs are able to work with the women in the villages that they live in, both the residents and the workers benefit, and this process can potentiate better health outcomes within the community. Health programming in India continues to be costly [19]. The study paves the way for future research studies to study efficient, and cost-effective, mechanisms to improve access to health in a community. In particular, we believe that CHWs can be an empathetic linkage between health systems and a rural population, whose diverse cultural and linguistic makeup can directly affect their typical usage of health care. Additionally, normalizing the use of technology will make it easier for CHWs to connect rural residents with health professionals, and for there to be more electronic records available for a given individual. Given the results of this study, it is feasible to incorporate CHWs into programs that advance scientific discoveries and promote preventive health measures. In summary, this feasibility project uses modern data technologies blended with community human resources to improve awareness and access to health care in a maternal and child health population in rural India.

## Supporting information

S1 Appendix Notification messaging. (DOCX)
