# Supplementary material for: Using community health workers to refer pregnant women and young children to health care facilities in rural West Bengal, India: A prospective cohort study
Source: PLoS One. 2018 Jun 21;13(6):e0199607. doi: 10.1371/journal.pone.0199607 (PMC6013192; doi:10.1371/journal.pone.0199607)
Supplement: S1 Appendix — (DOCX) [file pone.0199607.s001.docx]

**S1 Appendix. Notification messaging.**

*For pregnant women blood pressure below 110/70 mm Hg*

Your blood pressure is low which may cause you to have dizziness, light-headedness and even fainting. You need to consult a doctor, eat good food (fish, eggs, meat, milk etc.) and till you consult a doctor you must do the following - sit or lie down if you feel dizzy to avoid falls, avoid standing up too fast from seated or lying down position and lie on the left side to increase blood flow to your heart.

*For pregnant women who were anemic*

You have anemia that is less blood in your system. This may cause you to feel tired, dizzy, weak, breathless, have palpitations etc. This may adversely affect your child and you during childbirth. You need to consult your doctor immediately. Have your blood checked for anemia every two weeks. And till you visit your doctor, have food which will improve your hemoglobin like, meat, eggs, milk and milk products, chhola, dal, spinach, green peas, soyabean, beet, fruits such as orange, guavas etc. You may need to take some medicines to improve your blood. Consult your doctor immediately.

বিজ্ঞপ্তি বার্তা

গর্ভবতী মহিলাদের ক্ষেত্রে রক্তচাপ ১১০/৭০ মিমি এইচ.জি

আপনার রক্তচাপ কম থাকায় আপনি হাল্কা মাথা ঘোরা, দুর্বল অনুভব এবং অজ্ঞান ও হয়ে যেতে পারেন। আপনার একজন ডাক্তাররের পরামর্শ নেওয়া প্রয়োজন, ভালো খাওয়ার (মাছ, ডিম, মাংস, দুধ প্রভৃতি) এবং যতক্ষণ না আপনি ডাক্তার দেখাচ্ছেন আপনার এইগুলি অবশ্যই করণীয় – পড়ে যাওয়া থেকে বাঁচতে তৎক্ষণাৎ শুয়ে বা বসে পড়ুন যদি আপনার মাথা ঘোরে, বসে থাকা বা শুয়ে থাকা অবস্থা থেকে কখনই তাড়াহুড়ো করে দাঁড়াতে যাবেন না এবং শোওয়ার সময় বাঁ দিক করে শোবেন যাতে হৃৎপিণ্ডে রক্ত চলাচল বৃদ্ধি পায়।

গর্ভবতী মহিলা যারা রক্তাল্পতায় ভুগছে

আপনি অ্যানিমিয়ায় ভুগছেন, তার মানে আপনার শরীরে কম রক্ত রয়েছে। এর জন্য আপনি ক্লান্তবোধ করবেন, মাথা ঘোরাবে, শ্বাসপ্রশ্বাসে কষ্ট হবে, বুক ধড়ফড় ইত্যাদি করবে। এইগুলি আপনাকে ও জন্মের সময় আপনার সন্তানকে প্রভাবিত করতে পারে। আপনার এক্ষুনি ডাক্তারের সাথে আলোচনা করা উচিৎ। প্রতি দু সপ্তাহে আপনি রক্তাল্পতার জন্য রক্ত পরীক্ষা করান। এবং যতক্ষণ না আপনি ডাক্তার দেখাচ্ছেন, সে সব খাওয়ার খান যাতে হিমোগ্লোবিন বৃদ্ধি পায় মাংস, ডিম, দুধ, দুগ্ধপদার্থ, ছোলা, ডাল, সবুজ শাকসব্জি, সবুজ মটর, সয়াবিন, বিট, ফলমূল যেমন কমলালেবু, পেয়ারা ইত্যাদি। আপনার প্রয়োজন হতে পারে কিছু ওষুধ নিয়ে রক্তের পরিমানে উন্নতি করার জন্য। শীঘ্রই আপনার ডাক্তারের সাথে যোগাযোগ করুন।

*For children with abnormal anthropometric measurements*

We have examined your baby and have measured your baby’s height, weight, head circumference and mid-arm circumference to make sure that your baby is growing well and is healthy. However, we noticed certain things which we need to keep you informed about.

Your baby’s ………………..(can site the abnormalities). This is only a one-time measurement and may not mean anything. Also, temporary ups and downs from your baby's percentile curve are quite normal and usually not a cause for concern.   

Ups might happen with a growth spurt and temporary downs might happen after a bout of illness for example after a bout of diarrhea. You need to consult a doctor who is going to keep an eye on your baby. If your doctor notices that over time your baby is not keeping up with his/her curve, the doctor might investigate to see if something is preventing your baby from growing well.  If the baby is on solid foods, the doctor might ask questions about the baby's stool to determine if he/she is able to digest the food well. Your doctor will also give you tips on how to help your baby put on weight and eat well. 

Since you are concerned about your baby's growth, it was our responsibility to inform you of these current issues that we noticed. It is best to consult your doctor. Your doctor will most probably reassure you that all is well, or if the need arises, advise you on the proper next steps. 

Until you see your doctor, ensure that your child is fed well. Staple homemade food comprising of cereal-pulse mixture (Khichri) in the ratio 2:1 s fine but they must be made energy dense and protein-rich. Easily available, cost-effective seasonal fruits, green vegetables, milk and milk products, pulses/legumes, fish, eggs, oil/butter, sugar/jaggery must be added to the staples.

*For children with low Ages and Stages Questionnaire (ASQ) scores*

In our examination of the things your baby can do, we found areas that were behind. This may not be anything to worry about, but it is advisable that you consult your baby’s doctor, who can monitor this and advise you further about proper next steps. Until you see your doctor, encourage your child to copy sounds and words, continue to play and interact with your child.

অস্বাভাবিক আন্থ্রোপমেত্রিক পরিমাপসহ শিশুদের জন্য

আমরা আপনার শিশুর পরীক্ষা করেছি এবং তার উচ্চতা, ওজন ও ,মাথার পরিধি কত তা পরিমাপ করেছি, নিশ্চিত করতে যে আপনার সন্তান সম্পূর্ণ সুস্থ এবং সুসাস্থের অধিকারী। যদিও আমরা কিছু জিনিস লক্ষ্য করেছি যা আপনাকে জানিয়ে রাখা প্রয়োজন। আপনার শিশুর....................................................................

(অস্বাভাবিকতাগুলো বলুন)। এটি শুধুমাত্রই একবারের পরিমাপ, এবং এর আলাদা কোনও মানে নাও হতে পারে। এছাড়াও আপনার শিশুর পার্সেন্টাইল কার্ভের অস্থায়ী ওঠা নামা খুবই সাধারন এবং তা নিয়ে চিন্তিত হওয়ার কোনও প্রয়োজন নেই।

ওঠাগুলি স্থান নেবে অনেকটা দৌড়ঝাপের পরে এবং ক্ষণস্থায়ী নামা গুলি স্থান নেবে একটি রোগভোগ পর্যায়ের পরে, যেমন ডাইরিয়া হওয়ার পরে। আপনার অবশ্যই দরকার একজন ডাক্তার দেখান প্রয়োজন যে আপনাকে আপনার সন্তানের স্বাস্থ্য সম্পর্কে সচেতন করবে। যদি ডাক্তারবাবু লক্ষ্য করেন যে আপনার সন্তান অনেকদিন ধরেই তার পার্সেন্টাইল কার্ভটিকে উঁচুতে ওঠাতে পারছে না তাহলে ডাক্তারবাবু অবশ্যই পরীক্ষা করে দেখবেন যে কোনও কিছু আপনার সন্তান কে সঠিক বৃদ্ধির হাত থেকে আটকে রাখছে কিনা। শিশুটি যদি শক্ত খাওয়ার খায়, সেক্ষেত্রে ডাক্তারবাবু শিশুটির মল সম্পর্কে নানান প্রশ্ন করতে পারে এটা নির্ধারণ করতে যে শিশুটি ঠিক ভাবে খাওয়ার হজম করতে সক্ষম। আপনার ডাক্তারবাবু আপনাকে বাচ্চাটির ওজন বৃদ্ধি ও তাকে ভালো করে কিভাবে খাওয়াতে হবে সেই সম্পর্কে টিপস দেবে।

যেহুতু আপনি আপনার সন্তানের স্বাস্থ্য সম্পর্কে চিন্তিত, এটা আমাদের কর্তব্য আপনাকে জানানো যে সমস্যাগুলি আমরা লক্ষ্য করেছি। তাই ডাক্তারের সাথে পরামর্শ নেওয়া অবশ্যই জরুরী। আপনার ডাক্তার সম্ভবত আপনাকে আসস্থ করবে যে সব ঠিক রয়েছে, অথবা যদি প্রয়োজন দেখা দেয় তাহলে পরবর্তী পদক্ষেপ সম্পর্কে অবশ্যই সচেতন করবে।

কম বয়স ও পর্যায়ের শিশুদের প্রশ্নাবলির স্কোর

আমাদের পরীক্ষায় আমরা শিশুটির পিছিয়ে পড়া অংশ গুলি দেখার চেষ্টা করি। এটি চিন্তিত হওয়ার বিষয় নয়, কিন্তু এটি আবেদন করা হচ্ছে যে আপনার বাচ্চার ডাক্তারের থেকে পরামর্শ নিতে, যে এই বিষয়টি পর্যবেক্ষণ করবেন ও আপনাকে পরবর্তী পদক্ষেপ সম্পর্কে অবহিত করবে, যতক্ষণ পর্যন্ত না আপনি ডাক্তারের পরামর্শ নিচ্ছেন আপনার সন্তান কে মুখ দিয়ে আওয়াজ ও শব্দ বের করার উৎসাহ দিন, তার সাথে খেলাধুলো করুন এবং আরও বেশি করে যোগাযোগ গড়ে তুলুন।
